# Supplementary material for: Investigation of MO Adsorption Kinetics and Photocatalytic Degradation Utilizing Hollow Fibers of Cu-CuO/TiO2 Nanocomposite
Source: Materials (Basel). 2024 Sep 23;17(18):4663. doi: 10.3390/ma17184663 (PMC11434048; doi:10.3390/ma17184663)
Supplement: Supplementary file 1 [file materials-17-04663-s001.zip › materials-3187962-supplementary.pdf]

Investigation of MO adsorption kinetics and photocatalytic degradation utilizing hollow fibers of Cu-CuO/TiO_2 nanocomposite

George V. Theodorakopoulos [1,2,*], Sergios K. Papageorgiou [1], Fotios K. Katsaros [1], George Em. Romanos [1,*] and Margarita Beazi-Katsioti [2]

1 Institute of Nanoscience and Nanotechnology, National Center for Scientific Research "Demokritos", 15341 Agia Paraskevi, Athens, Greece
2 School of Chemical Engineering, National Technical University of Athens, Zografou Campus, 9 Iroon Polytechniou Street, 15772 Zografou, Athens, Greece
* Correspondence: g.theodorakopoulos@inn.demokritos.gr; g.romanos@inn.demokritos.gr

S1. Characterization results

SEM and EDS analysis

The SEM analysis confirms the rigid structure and proper shaping of the developed composite HFs. The wall thickness varies between 130 and 150 μm, exhibiting a fairly uniform texture and significant surface roughness, with an outer diameter of approximately 700 μm. The residual carbon formed during pyrolytic sintering serves as a binding agent between TiO_2 nanoparticles, facilitating the development of a loosely structured network (Figure S1). This network is composed of interconnected, fibril-like branches with diameters ranging from 12 to 20 nm, contributing to the material's porous architecture. The carbon binder plays a crucial role in stabilizing the nanoparticle framework, ensuring the nanoparticles remain connected while preserving a relatively open and loose configuration. Additionally, the voids between the nanoparticles and aggregates exhibit an average size ranging from 20 to 100 nm, which is consistent with the results obtained from the LN_2 porosimetry measurement. The TiO_2 nanoparticles are evenly dispersed throughout the composite HF matrix. The beneficial effect of copper nanoparticles, both metallic and CuO, in enhancing the sample's porosity is also clearly observed. SEM micrographs reveal a notably high density of voids (pores) within the sample, further supporting this increase in porosity.

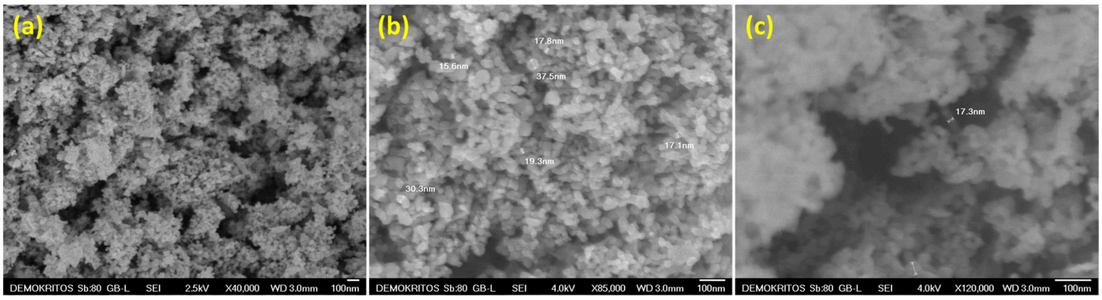

Figure S1. SEM images of the internal surface of the Cu-CuO/TiO_2 nanocomposite at magnifications of **(a)** 40,000x, **(b)** 85,000x, and **(c)** 120,000x [62].

The composition and dispersion properties of the Cu-CuO/TiO_2 nanocomposite was analyzed using EDS nanoanalysis. The average copper content was determined to be 3.1%, with values ranging from 2.6% to 3.5% across 10 different areas, which aligns well with the reported sorption capacity of alginate [62]. Figure S2 also illustrates the elemental distribution within the sample, confirming that all elements, including copper, are evenly dispersed, thereby verifying the material's homogeneous nature.

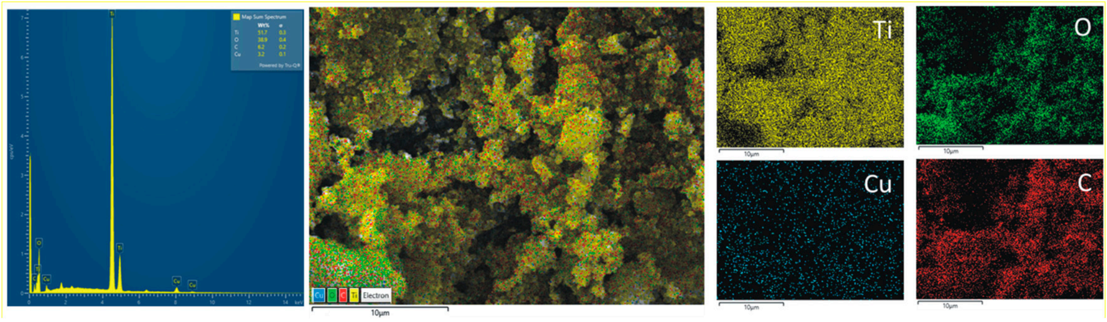

Figure S2. EDS spectrum and elemental mapping of the Cu-CuO/TiO_2 nanocomposite [62].

XRD analysis

The XRD patterns of both the Cu-CuO/TiO_2 nanocomposite and the Degussa P25 TiO_2 (for comparison) are shown in Figures S3a. The developed nanocomposite contains both anatase (2θ = 25.3, 37.0, 37.9, 38.7, 48.1, 54.0, and 55.1) and rutile (2θ = 27.5, 36.1, 41.3, 44.1, and 56.6) phases. Moreover, Bragg reflections corresponding to residual carbon and zero-valent metallic Cu are visible in the XRD diffractograms, while no peaks for CuO could be identified due to the complexity of the spectrum. The mean size of the metallic copper nanoparticles was calculated to be approximately 20 nm using Scherrer's formula at 43.3°.

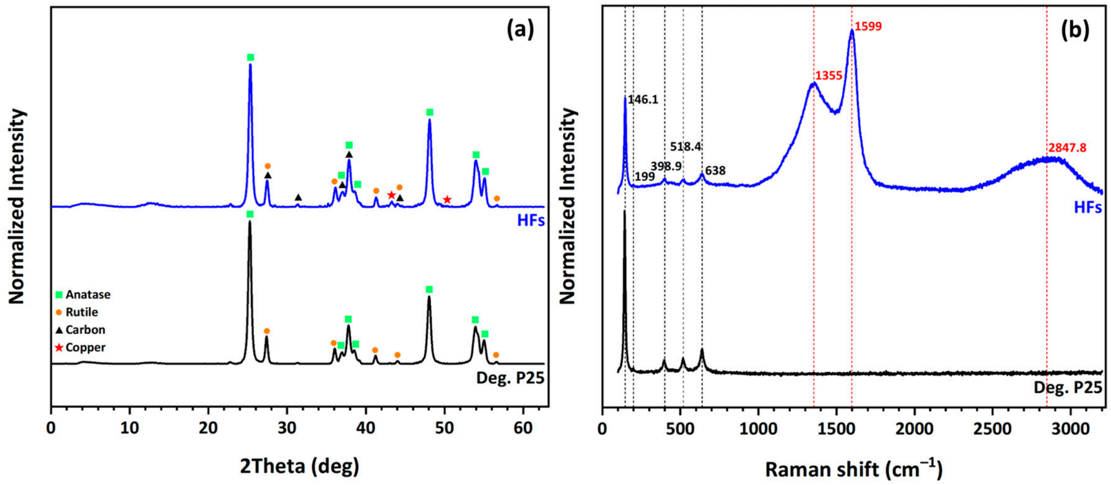

Figure S3. (a) XRD patterns and **(b)** Raman spectra of the Cu-CuO/TiO_2 nanocomposite in HF form compared to Degussa P25 TiO_2.

Raman analysis

In addition to the XRD analysis, Raman spectroscopy was used to identify the TiO_2 phases and detect residual carbon. The Raman bands for anatase were observed at approximately 146 (E_g), 199 (E_g), 399 (B_{1g}), 518 (a superposition of A_{1g} and B_{1g} modes), and 638 (E_g) cm^{-1} in the Cu/TiO_2 nanocomposite, with slightly shifts compared to the Degussa P25 TiO_2 (Figure S3b). Furthermore, the presence of a carbonaceous phase was confirmed after the nanocomposite's thermal treatment, indicated by the graphitic D and G bands at 1355 and 1600 cm^{-1}, respectively. The D band signals sp^2-hybridized carbon structures, while the G band corresponds to hexagonal graphitic networks. The 2D band, observed around 2850 cm^{-1}, as a second order (or overtone) of the D band, is typically employed to determine the thickness of graphitic layers. In this case, its asymmetry and large full width at half maximum (FWHM) suggest a multi-layered graphitic structure. The intensity ratio of the D to G bands (I_D/I_G) was calculated to be 0.6, indicating a moderate level of defects. While the graphitic domains remain relatively well-ordered, some defects, such as vacancies, dislocations, or edges, are present, indicating a partially disordered yet graphitic structure.

S2. Evaluation of MO adsorption capacity

Langmuir model

$$q_e = \frac{q_m \times b \times C_e}{1 + b \times C_e} \tag{S1}$$

where q_e (mg/g) is the amount of adsorbate adsorbed per unit mass of adsorbent, q_m is the maximum monolayer adsorption capacity (mg/g), C_e (mg/L) is the equilibrium concentration in the liquid phase, and b is the Langmuir adsorption constant (L/mg) [69].

$$R_L = \frac{1}{1 + b \times C_0} \tag{S2}$$

where C_0 (mg/L) is the initial MO concentration. The value of R_L indicates the type of the isotherm to be either unfavorable ($R_L > 1$), linear ($R_L = 1$), favorable ($0 < R_L < 1$), or irreversible ($R_L = 0$).

Freundlich model

$$q_e = K_F \times C_e^{\frac{1}{n}} \tag{S3}$$

where q_e (mg/g) is the amount of adsorbate adsorbed at equilibrium (mg/g), K_F is the Freundlich adsorption constant (L/g) representing the adsorption capacity, C_e is the equilibrium concentration of the adsorbate (mg/L), and n is the heterogeneity factor related to the adsorption intensity of the adsorbent. The magnitude of 1/n gives an indication of the favorability of adsorption (similar to the aforementioned R_L parameter). If the value of n = 1, the adsorption is linear; if n < 1, the adsorption is chemical, and if n > 1, the adsorption is a favorable physical process [72].

Sips model

$$q_e = \frac{q_m \times (b \times C_e)^{\frac{1}{n}}}{1 + (b \times C_e)^{\frac{1}{n}}} \tag{S4}$$

where q_m (mg/g) can be related to the total number of binding sites, b (L/mg) is the median association constant, and 1/n is the heterogeneity factor [73]. Values for 1/n close to zero indicate heterogeneous adsorbents, while values closer to or 1 indicate a material with relatively homogenous binding sites. In such cases, the Sips model is simplified to the Langmuir one.

Temkin model

$$q_e = \frac{R \times T}{b_T \times \ln(K_T \times C_e)} \tag{S5}$$

where q_e is the adsorbed dye amount on adsorbent at equilibrium (mg/g), C_e is the equilibrium concentration in solution (mg/L), R is the universal gas constant (8.314 J/mol·K), T is the absolute temperature (K), K_T is the equilibrium binding constant (L/g), and b_T is the variation of adsorption energy (kJ/mol) (in the linear form, the Temkin constant $B_1 = \frac{R \times T}{b_T}$ is also represented) [74].

Dubinin-Radushkevich (D-R) model

$$q_e = q_s \times e^{(-B_{DR} \times \varepsilon^2)} \tag{S6}$$

where B_{DR} and ε can be correlated as follows:

$$E = \frac{1}{(2 \times B_{DR})^{0.5}} \text{ and } \varepsilon = R \times T \times \ln\left(1 + \frac{1}{C_e}\right) \tag{S7}$$

where q_e is the adsorbed amount at equilibrium per unit weight of adsorbent (mg/g), C_e is the adsorbate equilibrium concentration (mg/L), q_s is the theoretical isotherm saturation capacity (mg/g), ε is the Polanyi potential, B_{DR} is the Dubinin–Radushkevich constant (mol^2/kJ^2), R is the universal gas constant (8.314 J/mol·K), T is the absolute temperature (K), and E is the mean free adsorption energy per molecule of adsorbate (kJ/mol). The value of adsorption energy also offers insights into the nature of the adsorption process [75].

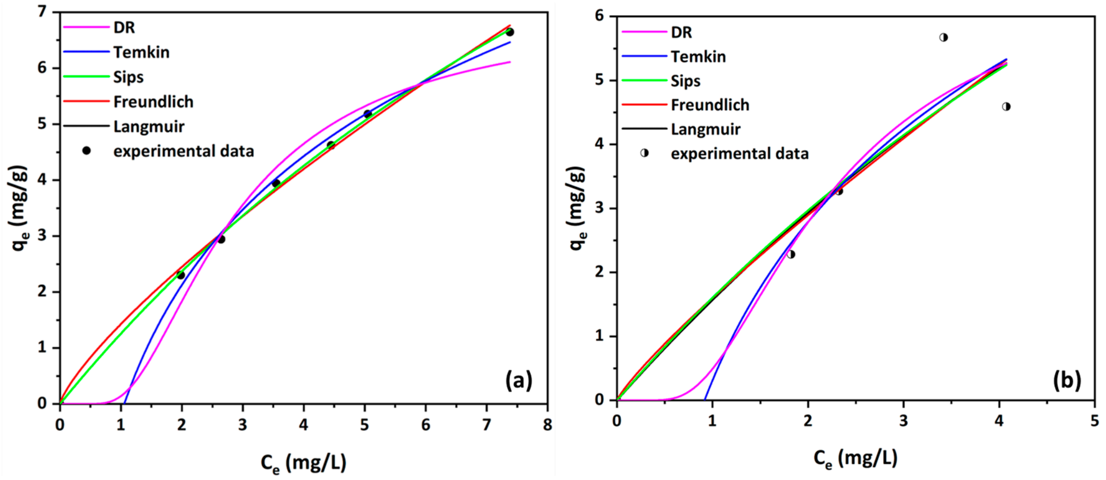

Figure S4. Experimental isotherms of MO adsorption and non-linear fitting of five isotherm models in **(a)** O_2-saturated and **(b)** O_2-depleted solutions (natural pH, 25 °C).

Table S1. Parameter data from the adsorption isotherm equations for MO uptake onto the Cu-CuO/TiO_2 nanocomposite in O_2-saturated and O_2-depleted solutions.

Langmuir	Freundlich	Sips	Temkin	D-R
$q_{mo} = 20.73$ / $q_{mi} = 22.80$	$K_{Fo} = 1.43$ / $K_{Fi} = 1.60$	$q_{mo} = 20.73$/ $q_{mi} = 20.07$	$b_{To} = 3.32$ / $b_{Ti} = 3.57$	$q_{so} = 6.96$ / $q_{si} = 6.86$
$b_o = 0.065$ / $b_i = 0.074$	$n_o = 1.28$ / $n_i = 1.17$	$b_o = 0.065$ / $b_i = 0.087$<br>$n_o = 1.00$/ $n_i = 1.00$	$K_{To} = 0.949$/ $K_{Ti} = 1.091$	$E_o = 0.62$ / $E_i = 0.75$
$R^2 = 0.999$/ $R^2 = 0.906$	$R^2 = 0.997$/ $R^2 = 0.902$	$R^2 = 0.999$ / $R^2 = 0.905$	$R^2 = 0.996$ /$R^2 = 0.921$	$R^2 = 0.972$ / $R^2 = 0.933$

Table S2. R_L and θ_{max} parameters for MO uptake in both O_2-saturated and O_2-depleted solutions.

$C_{initial}$ (mg/L)	R_L (-)		θ_{max} (-)	
	O_2-saturated	O_2-depleted	O_2-saturated	O_2-depleted
6.3	0.709	0.682	0.114	0.119
10	0.606	0.575	0.147	0.147
12	0.562	-	0.187	-
15	0.506	0.474	0.224	0.232
18	0.461	0.429	0.247	0.202
24	0.391	-	0.324	-

"The references in the supplementary information are cited in the main text, and their numbering matches that of the numerical list in the main text."
